# Determinants of diabetic retinopathy in Tikur Anbessa Hospital, Ethiopia: a case–control study

**DOI:** 10.1186/s40842-021-00128-5

**Published:** 2021-07-30

**Authors:** Kalid Seid, Temamen Tesfaye, Admasu Belay, Hayat Mohammed

**Affiliations:** 1 Department of Nursing, College of Health Science, Mizan-Tepi University, P.O. Box: 260, Mizan, SNNPR Ethiopia; 2 School of Nursing, Faculty of Health Science, Institute of Health, Jimma University, P.O. Box: 378, Jimma, Ethiopia; 3 Department of Medical Laboratory Science, College of Health Science, Mizan-Tepi University, P.O. Box: 260, Mizan, SNNPR Ethiopia

**Keywords:** Diabetic retinopathy, Determinants, Case–control, Ethiopia

## Abstract

**Background:**

Diabetic retinopathy is the most frequent complication of Diabetes Mellitus and remains the leading cause of preventable blindness. However, there are limited studies on the determinants of diabetic retinopathy in the study area as well in Ethiopia. Hence, this study aimed to assess the determinants of diabetic retinopathy among diabetic patients at Tikur Anbessa Hospital.

**Methods:**

An institution-based unmatched case–control study design was conducted at Tikur Anbessa Hospital from May 11 to June 26, 2020. Diabetic patients who developed retinopathy within 2 years were cases in the study. Patients who were free of retinopathy were controls in this study. Data were collected using a pretested interviewer-administered questionnaire, Topcon retinal examination, and a record review. The collected data were entered into Epi Data version 3.1 software, and analyzed using SPSS version 25. Binary logistic regression analysis was used to assess the determinants of diabetic retinopathy.

**Results:**

A total of 282 patients (142 cases and 140 controls) were included in the study. The mean age (± Standard deviation) for the cases and the controls were 50.6 (SD: ± 18.7) and 44.9 (SD: ± 17.65) respectively. Patients who had a glucometer at home (AOR = 0.048; 95% CI: 0.005–0.492), exercise adherence (AOR = 0.075; 95% CI: 0.007–0.84), diabetes duration < 5 years (AOR = 0.005; 95% CI: 0.00–0.10) and 5–10 years (AOR = 0.041; 95% CI: 0.003–0.57), health information on diabetic complications (AOR = 0.002; 95% CI: 0.00–0.042) and appointments every month (AOR = 0.004; 95% CI: 0.00–0.073) and every 3 months (AOR = 0.022; 95% CI: 0.002–0.23) were less likely to develop diabetic retinopathy. Participants who had poor glycemic control (AOR = 19.9; 95% CI: 2.34–168.69), systolic hypertension (AOR = 23.4; 95% CI: 2.56–215.36) and nephropathy (AOR = 17.85; 95% CI: 2.01–158.1), had a higher risk of developing diabetic retinopathy.

**Conclusions:**

Patients who had a glucometer at home, exercise adherence, diabetes duration < 10 years, health information on diabetic complications, and frequent follow-up had a preventive role. However, poor glycemic control, systolic hypertension, and nephropathy increase the risk of diabetic retinopathy. A concerted effort should be made to improve the health status of patients with Diabetes Mellitus, with particular emphasis on lifestyle modification practices to prevent diabetic retinopathy.

## Background

With the increasing prevalence of diabetes globally, diabetic retinopathy (DR) has become a severe threat to public health [1]. Globally, DR constitutes 5% of all blindness and affects 2 million people [2]. It is the most frequent complication of diabetes and remains the leading cause of preventable blindness [3, 4]. In addition, loss of productivity and quality of life in patients with DR results in additional socioeconomic burdens on the community [5].

Retinopathy is positively associated with all-cause mortality among adults, especially those with diabetes, and has also been shown to correlate with an increased risk of cardiovascular events [6]. Patients with DR had significantly worse scores on all scales of quality of life and life satisfaction than those without DR [7].

A global population census showed that of more than 32.4 million blind and 191 million visually impaired people, 0.8 million were blind, and 3.7 million were visually impaired due to DR [8]. Sadly, DR affects 30.2–31.6% of diabetes patients in Africa, especially in Sub-Saharan countries [9], and the presence and progression of DR are associated with significant increases in health care costs [10]. Yearly eye examinations reduce blindness by > 95%; however, compliance with annual eye examinations remains 50% or less in many areas [11]. In Ethiopia, the overall prevalence of DR among patients with DM is 19.48% [12]. The presence and severity of complications related to DR in the country are steadily increasing causes of premature death and disability [13].

The major risk factors (hyperglycemia, hyperlipidemia, and hypertension) have been extensively studied, and are strongly associated with DR [14, 15]. However, there is considerable variation in the consistency, pattern, and strength of these risk factors [4]. Some studies indicate that family history, prolonged DM duration, and glycemic control are well-established risk factors for DR [16, 17]. However, further studies are needed to confirm this relationship [18]. Therefore, apart from these factors, it is also essential to examine the effect of institutional factors on DR, which has not been well addressed by other quantitative studies.

In addition, there are limited studies on the determinants of diabetic retinopathy in the study area as well in Ethiopia. Moreover, the effects of significant determinant factors of DR are still discrepant and inconclusive among the Ethiopian population, which needs to be evaluated. Therefore, this study aimed to assess the determinants of DR among patients with Diabetes Mellitus at Tikur Anbessa Hospital, Addis Ababa, Ethiopia.

## Methods

### Study design, setting, and population

The institution-based unmatched case–control study design was conducted at the outpatient diabetic clinic of Tikur Anbessa Hospital, Addis Ababa, Ethiopia, from May 11 to June 26, 2020. The hospital receives approximately 400,000 patients referred from across Ethiopia annually. It is the largest teaching hospital in Ethiopia and provides a tertiary level referral treatment with 24 h emergency services, and it has different specialty clinics that provide follow-up services. The Diabetic clinic is the largest referral clinic for DM in the country, and provides services for more than 300 people per week regularly. Currently, there are more than 7000 diabetes mellitus patients on follow-up in the clinic.

For this particular study, the source population was diabetic patients on follow-up at the Tikur Anbessa Hospital diabetic clinic. The cases were diabetic patients who developed retinopathy within 2 years. Diabetic retinopathies had a history of diabetic retinopathy on the patient index card and had been diagnosed by ophthalmologists. In contrast, controls were diabetic patients free of retinopathy, which ophthalmologists decided during the data collection period. All adults (> 14 years) with DM who had no severe mental problems participated in the study.

### Sample size and sampling procedure

The sample size was determined using EPI-Info 7.1 statistical software. The assumptions for the sample size calculation were as follows: the proportion of poor glycemic control among cases [19], the minimum detectable odds ratio of 2.7, a confidence level of 95%, a power of 80%, a case to control the ratio of 1:1, and non-response rate of 5% which gives a final sample size of 284 (142 cases and 142 controls). Simple random sampling was used to recruit participants. The card numbers of the identified participants were coded for the cases and controls. After the codes of cases and controls, 223 cases and 1652 controls were entered into a computer-generated random number. A hundred forty-two cases and 142 controls were randomly selected.

### Data collection tools and procedures

The cases and controls were identified, and confirmed by physicians and ophthalmologists. For data collection, an interviewer-administered questionnaire, Topcon retinal examination, and record review were used. We conducted record reviews of patients with diabetes to identify cases and controls from records using a checklist. Cases and controls were recorded using the identification numbers. For the controls, an additional Topcon retinal examination was performed by an ophthalmologist. Retinal photographs were taken with a Topcon camera in a well darken room. Using photographic images, diabetic retinopathy was classified as present or absent in each eye. The ophthalmologist performed the classification, which was audited by a physician in the clinic during follow-up. After differentiating between cases and controls, data were collected from record review/patient identification cards and interviews with the study participants. Data were collected by two trained BSc nurses and 1 MSc nurse as supervisors. The questionnaire was prepared in English, translated to the Amharic language, and then back-translated to English by experts.

The questionnaire has four parts adapted from a study conducted at the Tikur Anbessa Hospital [20, 21]. It includes socio-demographic factors, behavioral factors, clinical factors, and institutional factors. The knowledge part has 07 items, and the attitude part has 05 items adopted from a study conducted in Saudi Arabia [22]. The Knowledge and attitude parts were tested for reliability with Cronbach alpha of 0.76 and 0.81, respectively. The trusting relationship was measured using the Revised Health Care Relationship (HCR-R) Trust Scale, which was tested for reliability with a Cronbach alpha of 0.91 [23]. For clinical factors, three results were obtained near the diagnosis of diabetic retinopathy for cases and near to data collection for free of diabetic retinopathy from patient medical records, and the average result was recorded.

### Measurements

An ophthalmologist measured diabetic retinopathy with any characteristic lesion on retinal camera examination, microaneurysms, hemorrhages, exudation, cotton wool spots, and/or new vessels [24]. Glycemic control was measured using fasting blood sugar (FBS). A DM patient with < 70 mg/dl and > 126 mg/dl average fasting blood sugar level of the last three records was coded as poor glycemic control, otherwise good glycemic control [25]. Body mass index was used to define underweight (BMI < 18.5), normal (18.5–24.9), overweight (25.0–30.0), and obesity (BMI > 30) in adults [26]. Hypertension was defined as systolic blood pressure (SBP) ≥ 140 mmHg, diastolic blood pressure (DBP) ≥ 90 mmHg or current use of antihypertensive medication. Two Measurements were taken from the left-arm 5 min apart in the sitting position. The average was recorded using a mercury sphygmomanometer with a cuff deflation rate of 2 mmHg [25]. Hyperlipidemia is defined as a total cholesterol level > 200 mg/dl or triglyceride level > 150 mg/dl [25]. Medication adherence was assessed if the patients took all their anti-diabetic medications in the last 7 days [27, 28]. Adherence to blood glucose testing at home was measured if the patient measured their blood glucose once a week [27, 28]. Adherence to diet was measured if the patient followed the recommended diet for more than 3 days in the last 7 days [27, 28]. Adherence to exercise was measured if the patient performed a 30-min activity prescribed by a physician for more than 3 days in the last 7 days [27, 28]. Alcohol consumption history was measured if the patient reported drinking any alcohol within the last 12 months [27, 28]. A positive attitude is defined as answered correctly more than the 50% score of attitude questions otherwise negative attitude [22]. ‘Excellent’ grade of knowledge if the score is higher than 75%; ‘Good’ if it is between 50 to 75%, and; ‘Poor’ if it is less than 50% [22]. A good trusting relationship with healthcare providers was measured as patients who answered correctly more than or equal to the mean score on the Revised Health Care relationship (HCR) trust scale, otherwise poor trusting relationships with healthcare providers [23].

### Data processing and analysis

The data were coded and entered into Epi-data 3.1, and exported to SPSS version 25 for analysis. Descriptive statistics such as frequency, percentage, standard deviation, and mean were used to summarize the data. In the current study, cases were coded as 1, and the controls were coded as 0. The exposure variables associated with DR were identified using a bivariate logistic regression analysis. Exposure variables with a *p*-value < 0.25 were considered for final multivariable logistic regression analysis. Statistical significance was set at *p*-value < 0.05, and exposure variables with a *p*-value < 0.05 were considered as determinants of DR. Model fitness was checked by Hosmer and Lemeshow (*P*-value = 0.698), omnibus likelihood test (*P*-value 0.00) with 96% model accuracy. The adjusted odds ratio (AOR) with a 95% confidence interval (CI) was computed to determine the strength of the association between the variables of interest.

## Results

### Socio-demographic characteristics

From a sample of 284 (142 cases and 142 controls), 282 (142 cases and 140 controls) participated in this study with a response rate of 99.3%. The mean age (± Standard deviation) for the cases and the controls had 50.6 (SD: ± 18.7) and 44.9 (SD: ± 17.65), respectively. Eighty (56.3%) cases and 60(42.9%) of the controls were male. Ninety-one (64.1%) of the cases and 86(61.4%) of the controls were married. Eighty-five (59.9%) cases and 82(58.6%) controls had urban residences. The majority (68.3%) of the cases and 93(66.4%) of the control’s monthly income had more than 1500 Ethiopian Birr (ETB) (Table 1).

**Table 1:** Socio-demographic characteristics of diabetic patients at Tikur Anbessa Hospital, Ethiopia, 2020 (*n* = 282)

**Variable**	**Category**	**Cases**	**Controls**
		**Number (%)**	**Number (%)**
**Age**	< 60 years	102 (71.8)	116 (82.8)
	≥ 60 years	40 (28.2)	24 (17.2)
**Sex**	Male	80 (56.3)	60 (42.9)
	Female	62 (43.7)	80 (57.1)
**Marital Status**	Single	16 (11.3)	37 (26.4)
	Married	91 (64.1)	86 (61.4)
	Divorced	19 (13.4)	10 (7.1)
	Widowed	16 (11.3)	7 (5.0)
**Residence**	Urban	85 (59.9)	82 (58.6)
	Rural	57 (40.1)	58 (41.4)
**Educational Status**	Unable to read and write	17 (12.0)	15 (10.7)
	Read and write	24 (16.9)	17 (12.1)
	Primary	18 (12.7)	15 (10.7)
	Secondary	23 (16.2)	20 (14.3)
	Diploma	30 (21.1)	34 (24.3)
	Degree and above	30 (21.1)	39 (27.9)
**Occupational Status**	Farmer	18 (12.7)	12 (8.6)
	Government Employee	36 (25.4)	65 (46.4)
	Self-employee	24 (16.9)	19 (13.6)
	Private business	36 (25.4)	27 (19.3)
	Non-employee	22 (15.5)	7 (5.0)
	Student	6 (4.2)	10 (7.1)
**Monthly Income**	≤ 1500 ETB	45 (31.7)	47 (33.6)
	> 1500 ETB	97 (68.3)	93 (66.4)

### Behavioral related characteristics

The majority of participants, 112(78.9%) cases and 84(60.0%) controls, had no glucometer at home. Most cases (90.1%) and 101 controls (72.1%) did not have a diet plan. Most cases (88.7%) and 90 controls (64.3%) did not adhere to the exercise regimen. More than half (52.1%) of the cases had poor knowledge of diabetic retinopathy, whereas 66(47.1%) of the controls had good knowledge. Regarding attitude, 94(66.2%) of the cases and 110(78.6%) of the controls had positive attitudes toward diabetic retinopathy (Table 2).

**Table 2:** Behavioral related characteristics of diabetic patients at Tikur Anbessa Hospital, Ethiopia, 2020 (*n* = 282)

**Variable**	**Category**	**Cases**	**Controls**
		**Number (%)**	**Number (%)**
**Glucometer at home**	Yes	30 (21.1)	56 (40.0)
	No	112 (78.9)	84 (60.0)
**Adherence to blood glucose measurement at home**	Adhered	11 (36.7)	26 (46.4)
	Not adhered	19 (63.3)	30 (53.6)
**Diet Plan**	Yes	14 (9.9)	39 (27.9)
	No	127 (90.1)	101 (72.1)
**Adherence to diet**	Adhered	2 (14.3)	11 (28.2)
	Not adhered	12 (85.7)	28 (71.8)
**Medication adherence**	Adhered	94 (66.2)	97 (69.3)
	Not adhered	48 (33.8)	43 (30.7)
**Exercise adherence**	Adhered	16 (11.3)	50 (35.7)
	Not adhered	126 (88.7)	90 (64.3)
**Alcohol**	Yes	54 (38.0)	28 (20.0)
	No	88 (62.0)	112 (80.0)
**Number of bottles per day**	≤ 2 bottle	30 (55.6)	17 (60.7)
	> 2 bottles	24 (44.4)	11 (39.3)
**Smoking**	Yes	30 (21.1)	37 (26.4)
	No	112 (78.9)	103 (73.6)
**Number of packets per day**	< 1 packet	25 (83.3)	28 (75.7)
	≥ 1 packet	5 (16.7)	9 (24.3)
**Knowledge of DR**	Poor	74 (52.1)	52 (37.1)
	Good	55 (38.7)	66 (47.1)
	Excellent	13 (9.2)	22 (15.7)
**Attitude toward DR**	Negative	48 (33.8)	30 (21.4)
	Positive	94 (66.2)	110 (78.6)

### Clinical related characteristics

Eighty-six (60.6%) of cases and 84 (60.0%) controls had Type II diabetes. More than half (52.1%) of cases had more than 15 years since diabetes diagnosis, but 82 (58.6%) of controls had less than 5 years. Regarding glycemic control, 109(76.8%) of cases had poor glycemic control, while 107(76.4%) of the controls had good glycemic control. Eighty-seven (61.3%) cases and 119 (85.0%) controls had no history of nephropathy. More than One-third of the 56(39.4%) cases and 32(22.9%) of the controls had obesity (Table 3).

**Table 3:** Clinical related characteristics of diabetic patients at Tikur Anbessa Hospital, Ethiopia, 2020 (*n* = 282)

**Variable**	**Category**	**Cases**	**Controls**
		**Number (%)**	**Number (%)**
**Type of DM**	Type I	56 (39.4)	56 (40.0)
	Type II	86 (60.6)	84 (60.0)
**Duration of DM**	< 5 years	12 (8.5)	82 (58.6)
	5–10 years	26 (18.3)	31 (22.1)
	11–15 years	30 (21.1)	15 (10.7)
	> 15 years	74 (52.1)	12 (8.6)
**Family history of DM**	Yes	42 (29.6)	43 (30.7)
	No	100 (70.4)	97 (69.3)
**Glycemic control**	Good	33 (23.2)	107 (76.4)
	Poor	109 (76.8)	33 (23.6)
**Hemoglobin level**	< 11 mg/dl	38 (26.8)	31 (22.1)
	≥ 11 mg/dl	104 (73.2)	109 (77.9)
**SBP**	< 140 mmHg	40 (28.2)	116 (82.9)
	≥ 140 mmHg	102 (71.8)	24 (17.1)
**DBP**	< 90 mmHg	84 (59.2)	89 (63.6)
	≥ 90 mmHg	58 (40.8)	51 (36.4)
**BUN**	< 6 mg/dl	47 (33.1)	23 (16.4)
	6–31 mg/dl	69 (48.6)	96 (68.6)
	> 31 mg/dl	26 (18.3)	21 (15.0)
**Creatinine**	> 1.2 mg/dl	73 (51.4)	44 (31.4)
	≤ 1.2 mg/dl	69 (48.6)	96 (68.6)
**Hyperlipidemia**	Yes	65 (45.8)	28 (20.0)
	No	77 (54.2)	112 (80.0)
**Neuropathy**	Yes	41 (28.9)	25 (17.9)
	No	101 (71.1)	115 (82.1)
**Nephropathy**	Yes	55 (38.7)	21 (15.0)
	No	87 (61.3)	119 (85.0)
**Treatment modality**	Injection/insulin	54 (38.0)	55 (39.3)
	Oral hypoglycemic	65 (45.8)	52 (37.1)
	Both insulin and oral hypoglycemic	16 (11.3)	15 (10.7)
	Diet or exercise	7 (4.9)	18 (12.9)
**History of cataract surgery**	Yes	16 (11.3)	11 (7.9)
	No	126 (88.7)	129 (92.1)
**BMI**	Underweight	18 (12.7)	14 (10.0)
	Normal	48 (33.8)	79 (56.4)
	Overweight	20 (14.1)	15 (10.7)
	Obesity	56 (39.4)	32 (22.9)

### Institutional related characteristics

One hundred and nineteen (83.8%) of the cases did not receive health information on diabetic complications, whereas 108(77.1%) of the controls had to obtain health information. Ninety-six (67.6%) cases and one-tenth (14.3%) of the controls were appointed every 6 months. Regarding trusting relationships with health care providers (HCPs), the majority (83.18%) of cases and 123(87.9%) of the controls had a good trusting relationship (Table 4).

**Table 4:** Institutional related characteristics of diabetic patients at Tikur Anbessa Hospital, Ethiopia, 2020 (*n* = 282)

**Variable**	**Category**	**Cases**	**Controls**
		**Number (%)**	**Number (%)**
**Getting health information on diabetic complication**	Yes	23 (16.2)	108 (77.1)
	No	119 (83.8)	32 (22.9)
**Difficulty in access to a diabetic care**	Yes	54 (38.0)	45 (32.1)
	No	88 (62.0)	95 (67.9)
**Type of difficulty**	The functionality of a screening device	27 (50.0)	10 (22.2)
	Distance to the facility	10 (18.5)	11 (24.4)
	Many people in the follow-up clinic	9 (16.7)	10 (22.2)
	Quality of the service	8 (14.8)	14 (31.1)
**Frequency of visit appointed**	Every month	21 (14.8)	55 (39.3)
	Every 3 months	25 (17.6)	65 (46.4)
	Every 6 months	96 (67.6)	20 (14.3)
**Trusting relationship with HCP**	Good	118 (83.1)	123 (87.9)
	Poor	24 (16.9)	17 (12.1)

### Determinants of diabetic retinopathy

In bivariate analysis, sex, presence of glucometer at home, diet plan, exercise adherence, alcohol consumption, knowledge of diabetic retinopathy, attitude toward diabetic retinopathy, duration of DM, glycemic control, systolic hypertension, creatinine level, hyperlipidemia, neuropathy, nephropathy, health information on diabetic complications and frequency of visits were found to be significantly associated with diabetic retinopathy. In multivariable logistic regression analysis, glucometer at home, exercise adherence, duration of diabetes, poor glycemic control, systolic hypertension, nephropathy, health information on diabetic complications, and frequency of visits were found to be independent determinants of diabetic retinopathy.

Patients who had a glucometer at home were 95.2% (AOR = 0.048; 95% CI: 0.005–0.492) less likely to develop DR than diabetic patients who had no glucometer at home. The proportion of respondents who adhered to exercise was 92.5% (AOR = 0.075; 95% CI: 0.007–0.84) less likely to develop DR than diabetic patients who did not adhere to exercise. Patients with diabetes duration less than 5 years were 99.5% (AOR = 0.005; 95% CI: 0.00–0.10) less likely to develop DR, and those between 5–10 years were 95.9% (AOR = 0.041; 95% CI: 0.003–0.57) less likely to develop DR than patients whose diabetes duration was more than 15 years. Individuals with poor glycemic control were 19 times (AOR = 19.9; 95% CI: 2.34–168.69) more likely to develop DR than those with good glycemic control.

Systolic hypertension was approximately 23 times (AOR = 23.4; 95% CI: 2.56–215.36) more likely to develop DR than their counterparts. Respondents who had nephropathy were approximately 17 times (AOR = 17.85; 95% CI: 2.01–158.1) more likely to develop DR than those who had no previous history of nephropathy. Patients who received health information on diabetic complications were 99.8% (AOR = 0.002; 95% CI: 0.00–0.042) less likely to develop DR than those who did not receive health information. Respondents who appointed every month had 96.6% (AOR = 0.004; 95% CI: 0.00–0.073) less likely to develop DR, and those patients who appointed every 3 months had 97.8% (AOR = 0.022; 95% CI: 0.002–0.23) less likely to develop DR compared to those who appointed for a visit every 6 months (Table 5).

**Table 5:** Multivariable logistic regression analysis of Diabetic Retinopathy in Tikur Anbessa Hospital, Ethiopia, 2020

**Variable**	**Category**	**Cases (*n* = 142)**	**Control (*n* = 140)**	**COR(95% CI)**	**AOR(95% CI)**	***P*-value**
**Sex**	Male	80(56.3)	60(42.9)	1.72(1.074–2.75)	3.65(0.47–28.01)	0.213
	Female	62(43.7)	80(57.1)	1	1	
**Glucometer at home**	Yes	30(21.1)	56(40.0)	0.40(0.23–0.68)	0.048(0.005–0.49	0.011*
	No	112(78.9)	84(60.0)	1	1	
**Diet Plan**	Yes	14(9.9)	39(27.9)	0.28(0.147–0.55)	0.33(0.032–3.47)	0.357
	No	127(90.1)	101(72.1)	1	1	
**Exercise Adherence**	Adhered	16(11.3)	50(35.7)	0.22(0.122–0.42)	0.075(0.007–0.84)	0.036*
	Not adhered	126(88.7)	90(64.3)	1	1	
**Alcohol**	Yes	54(38.0)	28(20.0)	2.45(1.43–4.19)	7.70(0.64–92.22)	0.107
	No	88(62.0)	112(80.0)	1	1	
**Knowledge of DR**	Poor	74(52.1)	52(37.1)	1	1	
	Good	55(38.7)	66(47.1)	0.58(0.35–0.96)	0.34(0.04–2.75)	0.317
	Excellent	13(9.2)	22(15.7)	0.41(0.19–0.89)	0.021(0.001–0.41)	0.011
**Attitude toward DR**	Negative	48(33.8)	30(21.4)	1.87(1.09–3.19)	0.83(0.127–5.43)	0.847
	Positive	94(66.2)	110(78.6)	1	1	
**Duration of DM**	< 5 years	12(8.5)	82(58.6)	0.024(0.01–0.05)	0.005(0.00–0.10)	0.001*
	5–10 years	26(18.3)	31(22.1)	0.136(0.06–0.30)	0.041(0.003–0.57)	0.018*
	11–15 years	30(21.1)	15(10.7)	0.324(0.13–0.77)	0.092(0.006–1.38)	0.085
	> 15 years	74(52.1)	12(8.6)	1		
**Glycemic control**	Good	33(23.2)	107(76.4)	1	1	
	Poor	109(76.8)	33(23.6)	10.7(6.17–18.58)	19.9(2.34–168.69)	0.006*
**SBP**	< 140 mmHg	40(28.2)	116(82.9)	1	1	
	≥ 140 mmHg	102(71.8)	24(17.1)	12.3(6.95–21.8)	23.4(2.56–215.36)	0.005*
**Creatinine**	> 1.2 mg/dl	73(51.4)	44(31.4)	2.3(1.421–3.75)	3.45(0.55–21.45)	0.183
	≤ 1.2 mg/dl	69(48.6)	96(68.6)	1	1	
**Hyperlipidemia**	Yes	65(45.8)	28(20.0)	3.37(1.98–5.73)	2.85(0.43–18.61)	0.273
	No	77(54.2)	112(80.0)	1	1	
**Neuropathy**	Yes	41(28.9)	25(17.9)	1.86(1.06–3.28)	3.68(0.308–44.22)	0.303
	No	101(71.1)	115(82.1)	1	1	
**Nephropathy**	Yes	55(38.7)	21(15.0)	3.58(2.018–6.35)	17.85(2.01–158.1)	0.010*
	No	87(61.3)	119(85.0)	1	1	
**Health information**	Yes	23(16.2)	108(77.1)	0.057(0.03–0.10)	0.002(0.00–0.042)	< 0.001*
	No	119(83.8)	32(22.9)	1	1	
**Frequency of visit appointed**	Every month	21(14.8)	55(39.3)	0.08(0.04–0.16)	0.004(0.00–0.073)	< 0.001*
	Every 3 months	25(17.6)	65(46.4)	0.08(0.041–0.15)	0.022(0.002–0.23)	0.002*
	Every 6 months	96(67.6)	20(14.3)	1	1	

^*^Significant at *P*-value < 0.05

## Discussion

In this case–control study of Diabetes Mellitus patients managed in Tikur Anbessa Hospital, several determinant factors for the occurrence of diabetic retinopathy were identified. In particular, patients who had a glucometer at home, exercise adherence, diabetes duration < 5 years and 5–10 years, health information on diabetic complications and being appointed every month and every 3 months had a reduced risk of DR. However, those with poor glycemic control, systolic hypertension and nephropathy had an increased risk of DR.

The current study showed that patients who had a glucometer at home had much fewer odds of having DR than those who did not. The possible reasons might be the ability of the patient to individualize their treatment regimen to obtain optimal blood glucose control. This individualized treatment allows for the detection and prevention of hypoglycemia and hyperglycemia. It plays a crucial role in normalizing blood glucose levels, which reduces the risk of long-term diabetic complications [25]. Therefore, maintaining tight control of blood sugar at home is the best way to avoid diabetes complications.

The current study also revealed that the odds of DR were significantly lower among patients who adhered to exercise than among those who did not adhere to exercise. This finding is in line with a case–control study conducted in the United Kingdom, which reported an inverse relationship between the severity of diabetic retinopathy and physical activity [29]. The possible reasons might be that exercise lowers blood glucose levels by increasing the uptake of glucose by body muscles and improving insulin sensitivity, decreasing the possibility of DR occurrence [25].

The current study found that the odds of DR increased in patients with a longer duration of diabetes. This finding is in line with previous studies conducted worldwide [21, 30–34], which reported that diabetes duration ≥ 10 years is the most significant risk factor for the occurrence of diabetic retinopathy.

Regarding glycemic control, individuals with FBS < 70Mmg/dl and > 126 mg/dl were much more likely to have DR than those with good glycemic control. This finding is consistent with previous studies conducted around the world [16, 30, 35–40], which reported that high levels of fasting blood glucose and HbA1c were associated with higher grades of retinopathy. This may be due to poor glycemic control, and diabetic blood-retinal barrier breakdown, which induces vascular leakage and macular edema [41].

The odds of DR were significantly higher among patients with systolic hypertension than among those without systolic hypertension. This finding is in line with previous studies conducted worldwide [20, 31, 33, 36, 37, 40, 42], which reported that DR increased with a mean systolic BP ≥ 140 mmHg. These findings underline the need for patient education among DM patients regarding the importance of BP control in preventing blindness from advanced DR.

The current study also revealed that the odds of DR were higher among patients with nephropathy. This finding is consistent with a case–control study conducted in Brazil [30] and Portugal [38], which reported a significant association between nephropathy and diabetic retinopathy. The possible reasons might damage the small blood vessels, which share the same pathogenesis as systemic microvascular dysfunction [43]. Therefore, optimizing blood-sugar control and tightly controlling blood pressure can reduce the risk of developing nephropathy because these diseases share the same pathological changes.

The current study showed that patients who received health information from health care providers about diabetic complications had lower odds of developing DR than those who did not receive health information. The possible reasons might be that when the patient receives health information on diabetic complications during follow-up, the patient’s awareness increases with inspiration to adhere to self-management goals [44].

Regarding the frequency of visits, respondents who were appointed every month and every 3 months had much fewer odds of having DR than those appointed for a visit every 6 months. This finding is in line with a cross-sectional study conducted in Addis Ababa, Ethiopia, which reported that participants who visited the diabetes clinic every month had lower odds of developing DR when compared to patients who visited the clinic every 6 months [21]. The possible reasons might be that frequent visits to the clinic enable health professionals and patients to know the progress of the disease and adjust the treatment option as early as possible before the occurrence of severe complications.

The strength of this study was that the case and control criteria were strictly defined to enrich the study for participants at the two extremes of the disease spectrum and minimize misclassification bias. The average baseline measurements for the cases and recent measurements of the controls were recorded. This study also assessed institutional factors that have not been addressed in previous studies.

However, this study had some limitations. Behavioral factors were collected from the current data, which may not be the same before the development of diabetic retinopathy. The lack of data on HbA1c data to measure glycemic control may affect the precision of the data. The use of self-report and review of the patient’s medical records for data collection may be subject to recall bias and missing data. The study sample is institution-based, limiting the generalizability of the results to the overall Ethiopian population.

## Conclusions

This study found that patients with a glucometer at home, exercise adherence, diabetes duration < 5 years and 5–10 years, health information on diabetic complications and appointments every month and every 3 months had a decreased risk of DR; however, patients with poor glycemic control, systolic hypertension and nephropathy had an increased risk of DR. In light of these findings targeted intervention should be designed at follow-up clinics to address the risk group and patients should maintain lifestyle modification practices to prevent diabetic retinopathy. Further studies using robust designs such as a prospective study by incorporating large samples should be conducted to explore more about the disease’s progression and assess related behavioral factors starting from the diagnosis. Further studies are needed to determine the relationship between the studied variables and specific subtypes of DR determined by severity or grade.

## Data Availability

Datasets obtained or analyzed during the current study are available from the corresponding author upon reasonable request.

## Abbreviations

AOR: Adjusted odds ratio
BMI: Body mass index
BUN: Blood urea nitrogen
CI: Confidence interval
COR: Crude odds ratio
DBP: Diastolic blood pressure
DM: Diabetes mellitus
DR: Diabetic retinopathy
ETB: Ethiopian birr
FBS: Fasting blood sugar
HCPs: Health care providers
HCR-R: Revised health care relationship
SBP: Systolic blood pressure
SD: Standard deviation
